# Impact of ^68^Ga-PSMA PET/MRI on the Accuracy of MRI-Derived Grading Systems for Predicting Extraprostatic Extension in Prostate Cancer

**DOI:** 10.3390/diagnostics15182405

**Published:** 2025-09-22

**Authors:** Lebriz Uslu-Beşli, Selahattin Durmaz, Aslıhan Onay, Barış Bakır, İclal Gürses, Sevda Özel-Yıldız, Çetin Demirdağ, Haluk Burçak Sayman

**Affiliations:** 1 Department of Nuclear Medicine, Cerrahpasa Faculty of Medicine, Istanbul University-Cerrahpasa, 34098 Istanbul, Turkey; hbsayman@yahoo.com; 2 Department of Radiology, Gaziosmanpasa Training and Research Hospital, 34255 Istanbul, Turkey; drselahattindurmaz@gmail.com; 3 Department of Radiology, Medical Faculty, TOBB University of Economics and Technology, 06510 Ankara, Turkey; aslionay@gmail.com; 4 Department of Radiology, Istanbul Faculty of Medicine, Istanbul University, 34093 Istanbul, Turkey; drbarisbakir@yahoo.com; 5 Department of Medical Pathology, Cerrahpasa Faculty of Medicine, Istanbul University-Cerrahpasa, 34098 Istanbul, Turkey; iclalgurses@gmail.com; 6 Department of Biostatistics, Istanbul Faculty of Medicine, Istanbul University, 34093 Istanbul, Turkey; sevda@istanbul.edu.tr; 7 Department of Urology, Cerrahpasa Faculty of Medicine, Istanbul University-Cerrahpasa, 34098 Istanbul, Turkey; cetindemirdag@gmail.com

**Keywords:** prostate-specific membrane antigen (PSMA), positron-emission tomography (PET), magnetic resonance imaging (MRI), prostate cancer (PCa), extraprostatic extension

## Abstract

**Objectives**: Accurate preoperative staging and prediction of extraprostatic extension (EPE) are critical for optimal surgical planning in prostate cancer (PCa). This study evaluated the diagnostic accuracy of ^68^Ga-PSMA PET for EPE assessment, compared it with the standardized multiparametric MRI (mpMRI)-derived EPE-grading system, and examined whether integrating semi-quantitative PSMA PET parameters improves diagnostic performance using hybrid PET/MRI. **Methods**: This retrospective, single-center study included treatment-naïve, biopsy-proven PCa patients who underwent ^68^Ga-PSMA-11 PET/MRI followed by radical prostatectomy. Diagnostic accuracy was assessed for clinical variables (PSA, ISUP grade), mpMRI features, mpMRI-derived EPE-grading system, visual PET findings, and semi-quantitative PET parameters (SUVmax, SUVmean, PSMA-tumor volume [PSMA-TV]). Optimal cut-offs were determined using the Youden index. Multivariate logistic regression and receiver operating characteristic (ROC) analyses were performed to compare the predictive value of clinical, mpMRI, or PET-derived variables, with histopathology as the reference standard. **Results**: Forty-five patients were included; EPE was histologically confirmed in 19 (42.2%). Predictors of EPE included capsular irregularity, neurovascular bundle asymmetry, curvilinear contact length ≥ 1.5 cm, seminal vesicle invasion, tumor size ≥ 14.25 mm, EPE grade ≥ 2, ISUP grade ≥ 3, overt EPE on PET, SUVmax ≥ 13.84, SUVmean ≥ 7.20, and PSMA-TV ≥ 1.40 cm^3^. The highest ROC performance (AUC = 0.890) was achieved by combining overt EPE on PET, SUVmax, and PSMA-TV. Incorporating PET parameters or tumor size into the EPE-grading system improved predictive accuracy. **Conclusions**: PSMA uptake in the primary tumor is an independent predictor of EPE. Integrating PSMA PET with mpMRI may provide additional information for preoperative EPE assessment.

## 1. Introduction

Accurate preoperative staging and risk assessment are important for therapy planning in prostate cancer (PCa) patients. While radical prostatectomy (RP) has excellent oncological outcomes, and is therefore frequently used for patients with localized PCa, it carries the risk of adverse effects such as urinary incontinence, usually resulting from the damage to the sphincter mechanism and erectile dysfunction due to neurovascular bundle injury. Nerve-sparing surgery was shown to reduce these RP-related side effects; however, it also increases the risk of positive surgical margins, which is more prominent in the presence of extraprostatic tumor extension (EPE) [1,2,3]. In contrast, a wider excision with removal of neurovascular bundle is recommended in the presence of EPE to reduce the risk of positive surgical margin and tumor recurrence [4]. Therefore, accurate prediction of EPE is crucial for proper surgical planning.

Multiparametric prostate MRI (mpMRI), which is commonly used for the selection of site of biopsy and preoperative local staging of PCa, was shown to be a better predictor of EPE compared to clinical-based models [5,6]. Pooled data from a meta-analysis showed a sensitivity and specificity of 0.57 (95% CI: 0.49–0.64) and 0.91 (95% CI: 0.88–0.93), respectively, for EPE assessment [7]. Several MRI features have been introduced as predictors of EPE, including capsular bulge or irregularity, overt EPE, obliteration of rectoprostatic angle, neurovascular bundle infiltration, seminal vesicle invasion, as well as MRI-derived measures such as radial tumor distance and length of tumor–capsule contact [8,9]. Hence, a standardized mpMRI-derived EPE-grading system was introduced for the preoperative prediction of the likelihood of EPE [10]. Nonetheless, the available evidence is still insufficient to make strong generalizable recommendations, regarding the choice between nerve-sparing and non-nerve-sparing surgery. In this context, preoperative risk stratification for EPE is best achieved by combining clinical parameters (e.g., serum prostate-specific antigen (PSA) level, PSA density, clinical stage, International Society of Urologic Pathologists (ISUP) grade group) with imaging-based indices such as Prostate Imaging-Reporting and Data System (PIRADS) score and mpMRI-derived features [11].

Gallium-68 (^68^Ga) prostate-specific membrane antigen (PSMA) positron-emission tomography (PET) imaging is a paradigm-changing imaging modality with higher sensitivity compared to mpMRI, contrast-enhanced computed tomography (CT) or other PET radiopharmaceuticals and excellent specificity in detection of nodal or organ metastases [11,12,13,14,15,16,17]. ^68^Ga-PSMA uptake was shown to be positively correlated with the tumor aggressiveness and higher Gleason scores, thus EPE is expected to be more frequent in tumors with high PSMA uptake [18,19]. However, the limited spatial resolution and partial volume effect of PET imaging is of concern for a reliable EPE assessment, and studies evaluating the value of ^68^Ga-PSMA PET compared with mpMRI in EPE assessment are contradictory. While some recent studies revealed higher sensitivity and improved surgical decision making for the nerve-sparing approach with ^68^Ga-PSMA PET compared to mpMRI in EPE assessment [20,21], there are also other studies where mpMRI outperformed ^68^Ga-PSMA PET in EPE assessment [22,23].

Our aim was to evaluate the diagnostic accuracy of ^68^Ga-PSMA PET for preoperative assessment of EPE and to compare it with the standardized mpMRI-derived EPE-grading system. We also investigated whether incorporating PSMA uptake metrics could enhance the predictive performance of the mpMRI-derived EPE-grading system in a hybrid PET/MRI setting.

## 2. Materials and Methods

This single-center retrospective study was conducted in accordance with the Helsinki Declaration, institutional guidelines and relevant regulations and was approved by the institutional clinical research ethics committee (7 August 2020/101824). Written informed consent was obtained from all participants.

### 2.1. Patient Population

**Figure 1:**
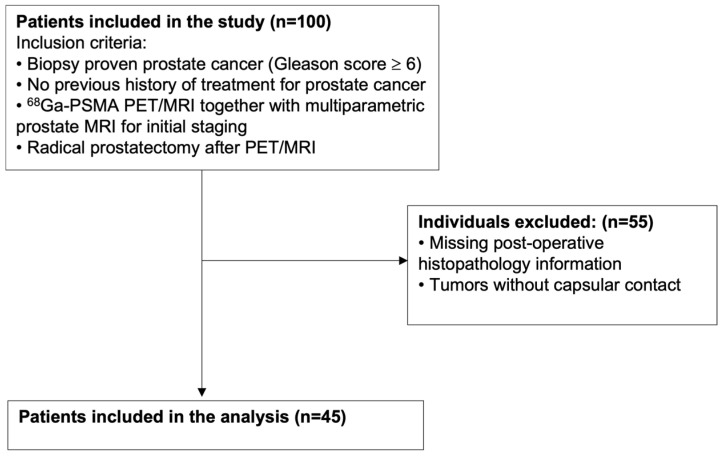
Flow chart showing patient selection process. n: Number of patients.

Between August 2017 and July 2022, we identified 100 patients with transrectal ultrasonography (TRUS) biopsy-proven, treatment-naïve clinically significant PCa (ISUP grade group ≥ 2) who underwent ^68^Ga-PSMA PET/MRI along with mpMRI for staging, followed by RP as a definitive treatment. Of these, 55 patients were excluded due to either absence of tumor–capsule contact on mpMRI or missing surgical histopathology data, resulting in a final study cohort of 45 patients. Age, serum PSA value, TRUS-biopsy results, and histopathology results of patients were collected. The study flow chart is shown in Figure 1.

### 2.2. ^68^Ga-PSMA PET/MRI

Pelvic ^68^Ga-PSMA-11 PET/MRI was performed on an integrated PET/MRI scanner (GE Signa PET/MRI, GE Healthcare, Waukesha, WI, USA) with a median uptake time of 106 ± 39 min and mean activity of 239.6 ± 60.0 MBq. ^68^Ga-PSMA-HBED-CC radiolabeling was performed using a fully automated radiopharmaceutical synthesis device, as previously described [24]. Patients were instructed to void before the onset of imaging. A single-bed PET emission scan was recorded with an acquisition time of 10 min over the pelvis. MpMRI was acquired simultaneously with the PET scan according to PIRADS v2.1 criteria. Attenuation correction for PET was performed using a vendor-based algorithm (VUEPoint FX time-of-flight reconstruction algorithm) which uses a robust, automated, MR-based attenuation correction (MRAC) procedure using DIXON sequence to create attenuation maps. Whole-body PET images, which were acquired before pelvic PET/MRI, were omitted from analysis.

### 2.3. Image Analysis

All images were analyzed using vendor-based workstation (GE AW Volume Share 7, GE Medical Systems, Buc, France) and readers were blinded to all clinical or histopathological findings. MpMRI images were independently evaluated by two radiologists (BB, 11 years of experience in mpMRI and SD, 5 years of experience). The following MRI features, as recommended in the recent PI-RADS guidelines and EPE-grading system proposed by Mehralivand et al., were assessed on T2-weighted (T2W) images to determine the likelihood of EPE: Capsular irregularity or bulge, overt EPE or invasion to other anatomical structures and asymmetry of the neurovascular bundle [10,25]. Presence of seminal vesicle invasion (SVI) was also assessed on mpMRI. Curvilinear contact length (CLL) with the capsule was measured using T2W images and ADC map, while tumor size was measured on ADC maps. Dynamic contrast-enhanced (DCE) MRI-based measurements were omitted, as early arterial enhancement was not present in all patients. The EPE-grading system was employed as follows: grade 0, no suspicion for EPE; grade 1, either CCL ≥ 1.5 cm or capsular irregularity and bulge; grade 2, both CCL ≥ 1.5 cm and capsular irregularity and bulge; grade 3, overt EPE visible at MRI or invasion of adjacent anatomic structures [10]. Common consensus was reached in case of disagreement between readers.

All PET images were reviewed independently by two experienced nuclear medicine physicians (LUB, 11 years of experience and HBS, >20 years of experience). The presence of overt EPE or SVI was assessed according to the European Association of Nuclear Medicine/Society of Nuclear Medicine and Molecular Imaging (EANM/SNMMI) criteria: EPE (miT3a) was defined as tumor activity extending beyond the prostate contour and SVI (miT3b) was defined as PET tumor activity extending into the seminal vesicle and/or separate focal activity in the seminal vesicle [26,27]. A final consensus was reached in case of disagreement between readers. Maximum and mean standardized uptake values (SUVmax and SUVmean) and PSMA tumor volume (PSMA-TV) with a threshold of 40% of the SUVmax were automatically calculated for the prostate lesion with increased PSMA uptake.

### 2.4. Histopathological Examination

Histopathology was considered the reference standard and whole prostatectomy specimens, including the seminal vesicles and ductus deferens were evaluated by an experienced uropathologist following the most recent World Health Organization (WHO) classification and ISUP recommendations at the time of prostatectomy [28,29,30,31]. The presence of EPE and SVI, as well as ISUP grade group, were retrospectively extracted from clinical pathology reports.

### 2.5. Statistical Analysis

Statistical analysis was performed using IBM SPSS version 28.0 and version 31.0 (IBM Corp., Armonk, NY, USA). Histopathological evaluation served as the reference standard for diagnostic confirmation. The level of significance was defined as *p* < 0.05.

Continuous variables, including serum PSA level, MRI-based tumor size measurement, SUVmax, SUVmean and PSMA-TV, were analyzed using receiver operating characteristic (ROC) curve analysis to determine an optimal cut-off point of predicted probability by maximizing the Youden index. Univariate logistic regression analysis was performed including the following variables: (1) MRI features: Capsular irregularity or bulge, presence of overt EPE or invasion to other anatomical structures, neurovascular bundle asymmetry, CLL ≥ 1.5 cm, SVI, tumor size ≥ cut-off point; (2) EPE-grading system: EPE grade ≥ 1, EPE grade ≥ 2, EPE grade ≥ 3; (3) ISUP grade group: ISUP grade ≥ 3, ISUP grade ≥ 4; (4) PET parameters: overt EPE; overt SVI; SUVmax ≥ cut-off point, SUVmean ≥ cut-off point, PSMA-TV ≥ cut-off point; and (5) PSA ≥ cut-off point. All variables were binary (positive or negative). Level of significance and diagnostic tests were assessed for each variable. Multivariate logistic regression analysis included significant variables from the univariate analysis to determine the independent association of each variable with EPE. Independent variables with *p* = 0.20 were also included in the multivariate analysis. Correlated variables were excluded from multivariate analysis. The models were structured to reflect both modality-specific (mpMRI-only or PET-only) and integrated (mpMRI plus PET) approaches. No automated variable selection methods were used; instead, the combinations were manually designed to compare the diagnostic performance of different imaging features in predicting EPE through ROC curve analysis. The following multivariate models were compared: (1) MRI-derived EPE grade and ISUP grade; (2) MRI-derived EPE grade and ISUP grade and tumor size on MRI; (3) PET features only; (4) MRI-derived EPE grade and PET features; (5) MRI-derived EPE grade and ISUP grade and PET features; (6) MRI-derived EPE grade and ISUP grade and tumor size on MRI and PET features.

A backward stepwise procedure was used to identify independent predictors of EPE. Area under the ROC curve (AUC) was compared between the multivariate models. To account for the increased risk of Type I error from multiple comparisons, we applied the Benjamini–Hochberg procedure to control the false discovery rate. P-values from the multivariate model comparisons were adjusted using this method, and significance was determined based on the corrected thresholds.

## 3. Results

### 3.1. Patients

Forty-five patients met the inclusion criteria and were included in the final analysis (Figure 1). The mean age was 65 ± 7 years (range: 53–76 years) and the mean serum prostate-specific antigen (PSA) level was 14.47 ± 17.17 ng/mL (range: 2.7–90.0 ng/mL). All patients underwent RP at a mean interval of 44 ± 29 days after PET/MRI acquisition. Final histopathology after RP revealed ISUP grade group 1 (n = 2, 4.4%), grade group 2 (n = 20, 44.4%), grade group 3 (n = 17, 37.8%), grade group 4 (n = 4, 8.9%), and grade group 5 (n = 2, 4.4%) prostate adenocarcinoma. EPE was confirmed by final histopathology after RP in 19 of 45 patients (42.2%). Patient characteristics are summarized in Table 1.

### 3.2. Prediction of EPE

The optimal diagnostic threshold using Youden index was 14.25 mm for MRI-based tumor size (Youden index = 0.520, sensitivity = 78.9% and specificity = 73.1%); 13.84 for SUVmax (Youden index = 0.449, sensitivity = 52.6% and specificity = 92.3%); 7.195 for SUVmean (Youden index = 0.425, sensitivity = 57.9% and specificity = 84.6%); 1.395 cm^3^ for PSMA-TV (Youden index = 0.601, sensitivity = 94.7% and specificity = 65.4%); and 0.8717 for logPSA (Youden index = 0.30, sensitivity = 68.4% and specificity = 61.5%).

Sensitivity, specificity, positive and negative predictive value, and accuracy of the diagnostic variables are given in Table 2. Among these variables, capsular irregularity or bulge, neurovascular bundle asymmetry, CLL ≥ 1.5 cm, SVI on MRI, tumor size ≥ 14.25 mm on MRI, EPE grade ≥ 1, EPE grade ≥ 2, overt EPE on PET; SUVmax ≥ 13.84, SUVmean ≥ 7.195, PSMA-TV ≥ 1.395 cm^3^ were statistically significant predictors of EPE (Table 3). Overt EPE on mpMRI and EPE grade ≥ 3 were both present in only nine patients; therefore, they were not included in the analysis.

**Figure 2:**
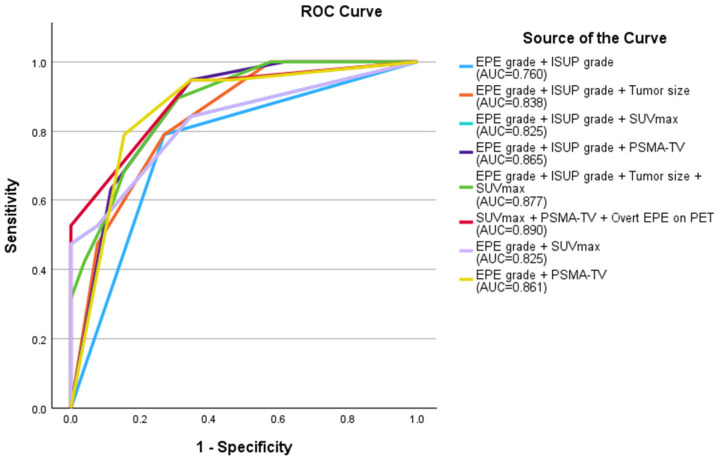
Receiver operating curve of the multivariate models compared. The combination of overt EPE on PET, SUVmax ≥ 13.84 and PSMA-TV ≥ 1.395 cm^3^ had the highest AUC of 0.890, although the difference from other models was not statistically significant (red curve). EPE: extraprostatic extension; ISUP: International Society of Urologic Pathologists; AUC: area under the curve; SUVmax: maximum standardized uptake value; PSMA-TV: prostate-specific membrane antigen tumor volume; PET: positron-emission tomography.

**Figure 3:**
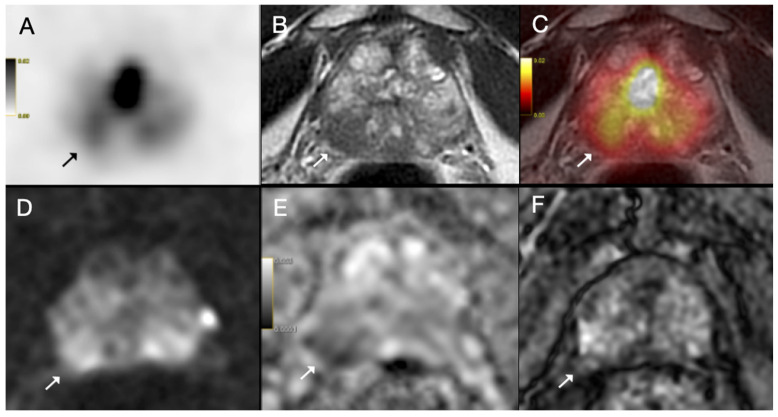
(**A**) Axial ^68^Ga-PSMA PET, (**B**) axial T2-weighted MRI, (**C**) fused PET/MRI, (**D**) diffusion-weighted image, (**E**) apparent diffusion coefficient map and (**F**) dynamic contrast-enhanced MRI images of a 70-year-old patient with prostate cancer. PI-RADS 5 tumor was detected on MRI in the right peripheral zone with prominent diffusion restriction and early arterial enhancement (arrow). Capsular bulging was present and capsular contact length was above 1.5 cm (EPE grade 2); however, PET image showed only mild PSMA uptake (SUVmax = 7.98). Postoperative histopathology confirmed ISUP grade group 2 prostate acinar adenocarcinoma without extraprostatic extension.

**Figure 4:**
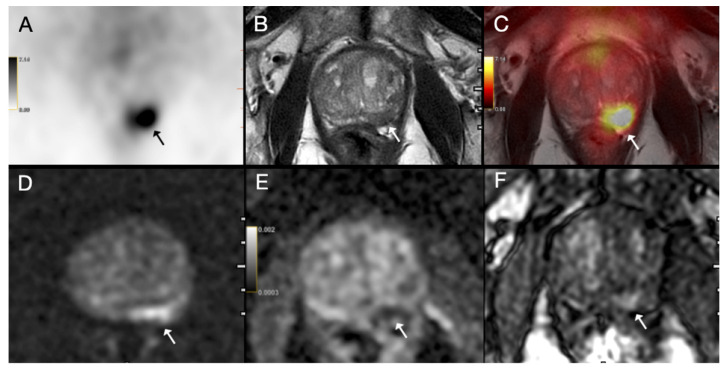
(**A**) Axial 68Ga-PSMA PET, (**B**) axial T2-weighted MRI, (**C**) fused PET/MRI, (**D**) diffusion-weighted image, (**E**) apparent diffusion coefficient map and (**F**) dynamic contrast-enhanced MRI images of a 71-year-old patient with prostate cancer. PI-RADS 5 tumor was detected on MRI in the left peripheral zone with prominent diffusion restriction and early arterial enhancement (arrow). MRI was negative for extraprostatic extension (EPE grade 0); however, PET image showed intense PSMA uptake (SUVmax = 13.88) (arrow). Postoperative histopathology confirmed ISUP grade group 4 prostate acinar adenocarcinoma with extraprostatic extension.

The following multivariate models were compared: (1) EPE grade ≥ 2 and ISUP grade ≥ 3; (2) EPE grade ≥ 2 and ISUP grade ≥ 3 and tumor size ≥ 14.25 mm; (3) overt EPE on PET and SUVmax ≥ 13.84 and PSMA-TV ≥ 1.395 cm^3^; (4) EPE grade ≥ 2 and SUVmax ≥ 13.84; (5) EPE grade ≥ 2 and PSMA-TV ≥ 1.395 cm^3^; (6) EPE grade ≥ 2 and ISUP grade ≥ 3 and SUVmax ≥ 13.84; (7) EPE grade ≥ 2 and ISUP grade ≥ 3 and PSMA-TV ≥ 1.395 cm^3^; (8) EPE grade ≥ 2 and ISUP grade ≥ 3 and tumor size ≥ 14.25 mm and SUVmax ≥ 13.84. ROC analysis of these multivariate models revealed that Model 3, which combined overt EPE on PET, SUVmax ≥ 13.84 and PSMA-TV ≥ 1.395 cm^3^ had the highest AUC of 0.890, although the difference from other models was not statistically significant (Figure 2, Figure 3 and Figure 4).

## 4. Discussion

Reliable preoperative detection of EPE is essential for optimizing surgery in PCa. Nerve-sparing RP decreases the risk of postoperative urinary incontinence and erectile dysfunction, but it also increases the risk of positive surgical margins and early biochemical recurrence in the presence of unrecognized EPE [32,33]. In contrast, overestimation of EPE can impair the quality of life. Despite being the current standard, mpMRI alone is not sufficiently accurate to guide nerve-sparing decisions; therefore, mpMRI findings are evaluated together with several clinical nomograms [8,9,11,34,35]. This study evaluated whether integrating semi-quantitative ^68^Ga-PSMA PET parameters into the mpMRI-derived EPE-grading system could improve the prediction of EPE and guide clinical decision making.

Among mpMRI-derived EPE-grading system features, capsular irregularity or bulge, neurovascular bundle asymmetry, CLL ≥ 1.5 cm and SVI on MRI were found to be statistically significant predictors of EPE in our cohort (Table 3). Overt EPE is also included in the EPE-grading system, however due to the limited number of patients with overt EPE in our cohort, it was not included in our univariate analysis. Similarly, while EPE grade ≥ 1 and grade ≥ 2 were significant predictors of EPE (*p* = 0.004 and *p* = 0.001, respectively), there were only nine patients with EPE grade ≥ 3, therefore it was also not included in the univariate analysis (Table 3). The sensitivity, specificity and accuracy were 78.9%, 73.1% and 75.6%, respectively, for EPE grade ≥ 2 and 31.6%, 88.5% and 64.4% for EPE grade ≥ 3 in our cohort. While Mehralivand et al. reported lower sensitivity and higher specificity of 61% and 81%, respectively, for EPE grade ≥ 2 patients, our sensitivity and specificity values were comparable for EPE grade ≥ 3 patients (31.6% and 88.5%, respectively, versus 30% and 96%, respectively) [10]. MRI features demonstrated the typical sensitivity-specificity trade-off. CCL ≥ 1.5 cm achieved the highest sensitivity (89.5%) but limited specificity (57.7%), whereas SVI on MRI and EPE grade ≥ 3 were highly specific (92.3% and 88.5%) but insensitive (52.6% and 31.6%, respectively) (Table 2). There is a substantial variation among the sensitivity values of mpMRI for detection of EPE in the literature (0–100%) [36]. The retrospective single-center study by Lee et al. incorporating 1045 PCa patients who had preoperative mpMRI reported sensitivity and specificity of 54.5% and 80.5%, respectively, for prediction of EPE [37]. The meta-analysis by de Rooij et al. incorporating 5681 patients from 45 studies on EPE detection, revealed a poor pooled sensitivity of 0.57 (95% CI 0.49–0.65) and an excellent pooled specificity of 0.91 (95% CI 0.88–0.93) for MRI [7].

^68^Ga-PSMA PET provided complementary functional information. All semi-quantitative PET parameters, including SUVmax, SUVmean and PSMA-TV, as well as visual PET assessment (i.e., overt EPE on PET) were found to be independent predictors of EPE (Table 3). SUVmax ≥ 13.84 yielded 92.3% specificity, while PSMA-TV ≥ 1.395 cm^3^ demonstrated 94.7% sensitivity and 94.4% NPV, indicating its potential to detect MRI-occult EPE (Table 2). These findings support the biological rationale that larger and more aggressive tumors are more likely to present with EPE [19,38,39]. Univariate logistic regression identified capsular irregularity (OR = 10.2), CLL ≥ 1.5 cm (OR = 11.6), SUVmax ≥ 13.84 (OR = 13.3), and PSMA-TV ≥ 1.395 cm^3^ (OR = 34.0) as strong predictors of EPE (Table 3). Among these, PSMA-TV had the highest odds ratio, confirming its independent role in EPE prediction.

In multivariate analysis, our best-performing model included only PET parameters, including overt EPE on PET + SUVmax ≥ 13.84 + PSMA-TV ≥ 1.395 cm^3^, which achieved AUC 0.890 (95% CI: 0.792–0.988) (Figure 2). Incorporation of the tumor size measurement or PET parameters to the EPE-grading system also yielded higher AUCs compared to EPE grade + ISUP grade only. Woo et al. recently published a study integrating ^18^F-DCFPyL PSMA PET/CT functional information with mpMRI, including the EPE-grading system proposed by Mehralivand et al., for preoperative prediction of EPE [40]. They reported higher AUC values for tumor size and CCL (on both mpMRI and PET) compared with the PRIMARY score, and a higher AUC for mpMRI-based CCL compared with SUVmax. There was no significant difference between AUCs of EPE grade, SUVmax and PSMA Likert scores (0.72, 0.65, and 0.62, respectively). In their study, integrating SUVmax with morphological MRI information yielded significantly higher sensitivity (80.4% vs. 66.7%) and similar specificity (81.2% vs. 87.5%) compared with mpMRI-based CCL. Spielvogel et al. also incorporated semi-quantitative PET and mpMRI parameters obtained from preoperative ^68^Ga-PSMA PET/MRI and established a machine-learning model with AUC 0.88 to predict EPE, which significantly outperformed conventional visual reads [41]. Our study extends this by providing quantitative cut-offs for SUVmax and PSMA-TV with immediate clinical applicability.

Although previous studies have reported inconsistent results regarding the performance of PSMA PET/CT and MRI in detecting EPE, several studies support the complementary roles of PSMA PET and mpMRI. A previous study comparing mpMRI with ^68^Ga-PSMA PET/MRI revealed AUC, sensitivity, and specificity as 0.66 vs. 0.73 (*p* = 0.19), 46% vs. 69% (*p* = 0.04), and 75% vs. 67% (*p* = 0.19), respectively in patient-based analysis for detection of EPE [42]. Woo et al. demonstrated pooled sensitivity 0.72 and specificity 0.87 for PSMA PET in EPE assessment, with PET/MRI outperforming PET/CT [43]. Brauchli et al. showed that the tumor–capsule interface on ^18^F-DCFPyL PSMA PET/CT predicts EPE with specificity comparable to mpMRI, especially in MRI-occult tumors [38]. Sonni et al. on the other hand reported that mpMRI had a higher AUC than ^68^Ga-PSMA PSMA PET/CT for detection of EPE (0.79 vs. 0.59, *p* = 0.002) [23]. Arslan et al. found lower sensitivity (56.2% vs. 62.5%) and higher specificity (82.6% vs. 60.8%) for mpMRI compared to ^68^Ga-PSMA PET/CT in their cohort [44]. In a recent study by Kivikallio et al., both ^18^F-PSMA PET/CT and mpMRI demonstrated low sensitivity but high specificity for detecting EPE, with no substantial difference in overall performance [45].

A recent meta-analysis revealed a diagnostic accuracy of 73% (95% CI: 64–82%) and 77% (95% CI: 69–85%) for PSMA PET/CT and PET/MRI, respectively for preoperative detection of EPE [46]. In all nine articles included in this analysis, EPE was assessed visually in a binary manner, which creates difficulties in the clinical practice and limits its interreader agreement due to partial volume effect and limited resolution of PET systems. Also, as opposed to mpMRI, established PET criteria to evaluate EPE are lacking for ^68^Ga-PSMA PET, which limits the reproducibility of the PET studies. Nevertheless, current EANM/SNMMI guideline recommends including SUVmax measurement in ^68^Ga-PSMA PET reports [26]. SUVmax was shown to have a strong correlation with the Gleason scores and was reported to be increased in patients with ISUP score > 2 and in patients with lymph node metastasis [19,47,48]. SUVmax was also shown to aid in discrimination of patients with significant prostate cancer: SUVmax cut-off value of 5.3 had a sensitivity of 85.9% and specificity of 86.21% with AUC = 0.893 for discriminating clinically significant prostate cancer from benign prostate disease [49]. Although several studies showed promising results for SUVmax measurement in ^68^Ga-PSMA PET images, SUVmax measurement repeatability and accuracy is a matter of concern as SUVmax values depend on several factors, such as amount of injected radiopharmaceutical activity and imaging time [50]. In our cohort, onset of pelvic PET/MRI scan was delayed in most of the patients due to longer whole-body PET acquisition time with PET/MRI compared to conventional PET/CT. Nonetheless, SUVmax, SUVmean and PSMA-TV values were predictors of EPE, which reflects higher SUVmax values in more aggressive tumors [19,47,48]. Also, PSMA-TV and SUVmax contribute specificity and high NPV in EPE assessment, which is critical in nerve-sparing decision making.

Artificial intelligence has growing interest in the field of medical imaging, as it can process high volume of datasets and detect subtle radiomic features. Machine learning models and deep learning architectures are used to identify complex imaging features and has the potential to predict EPE more accurately than conventional visual analysis [51,52]. Bian et al. compared radiomics model with the Mehralivand Grading System for prediction of EPE using ^18^F-PSMA PET/CT and found significantly higher AUC for radiomics model (75.8% vs. 66.8%) [53]. Yao et al. constructed a deep learning model integrating mpMRI and ^18^F-PSMA PET/CT for prediction of EPE and reported significantly higher AUC and sensitivity for deep learning-assisted EPE-grading scoring compared to radiologist EPE-grading scoring [54]. The multimodal imaging deep learning model had higher AUC compared to deep learning models with mpMRI alone or PSMA PET/CT alone.

To our knowledge, this is the first study to incorporate semi-quantitative PET parameters into the mpMRI-derived EPE-grading system to improve the diagnostic performance of preoperative imaging for prediction of EPE. This integrated approach may help better identify subtle EPE, potentially reducing the risk of unexpected positive margins and enhancing confidence in surgical planning, particularly in candidates for nerve-sparing prostatectomy.

As for the limitations of this study, retrospective design and modest sample size are the main limitations, which may introduce patient selection bias. We acknowledge that the relatively small sample size (45 patients) may limit the statistical power and generalizability of our findings and carries a risk of overfitting in the multivariate models. In addition, more than half of the patients initially included in this study were excluded due to missing pathology information or absence of capsule contact on MRI, which may bias the final cohort toward certain disease profiles. As this is a single-center study, reproducibility of the semi-quantitative parameters needs to be validated in prospective multi-center studies with larger patient populations. We used hybrid PET/MRI device, and the onset of pelvic PET imaging was delayed due to longer whole-body PET acquisition time compared to conventional PET/CT systems, therefore the semi-quantitative parameters need to be validated using PET/CT systems. The inclusion of patients with capsular contact may have increased the pretest probability of EPE, making the capsule sign appear more favorable than in daily practice, where more than one third of clinically significant cancers do not show capsular bulging. However, because preoperative prediction of EPE is crucial for surgical planning, we deliberately focused on patients with capsular contact, in whom prediction is often most relevant and clinically challenging.

## 5. Conclusions

Our study demonstrates that semi-quantitative ^68^Ga-PSMA PET metrics, including SUVmax, SUVmean and PSMA-TV, enhance preoperative EPE prediction when integrated with the mpMRI-based grading system, achieving AUC 0.890. This approach provides robust, quantitative guidance for nerve-sparing surgical planning, balancing oncological safety and functional preservation in prostate cancer.

## Figures and Tables

**Table 1:** Patient characteristics.

Number of patients	45
Age, years, mean ± SD	65 ± 7
PSA value at time of PET/MRI, mean ± SD	14.47 ± 17.17
ISUP grades obtained by histopathology, n (%)	
ISUP grade 1	2 (4.4)
ISUP grade 2	20 (44.4)
ISUP grade 3	17 (37.8)
ISUP grade 4	4 (8.9)
ISUP grade 5	2 (4.4)
Presence of extraprostatic extension at histopathology, n (%)	19 (42.2)
Presence of seminal vesicle infiltration at histopathology, n (%)	12 (26.7)
Tumor extent in the prostate, mean ± SD (%)	21.77 ± 25.43
Presence of lymphovascular invasion at histopathology, n (%)	12 (26.7)
Presence of lymph node metastasis at histopathology, n (%)	9 (20)
Presence of bone metastasis detected by imaging, n (%)	3 (6.7)
Presence of visceral organ metastasis detected by imaging, n (%)	0 (0)

SD, standard deviation; PSA, prostate-specific antigen; PET/MRI, positron-emission tomography/magnetic resonance imaging; ISUP, International Society of Urologic Pathologists; n, number of patients.

**Table 2:** Diagnostic test results for MRI features, EPE-grading system, clinical variables and ^68^Ga-PSMA PET features.

	Sensitivity (%)	Specificity (%)	PPV (%)	NPV (%)	Accuracy (%)
MRI Features					
Capsular irregularity or bulge	78.9 [54.4–94.0]	73.1 [52.2–88.4]	68.2 [52.2–80.8]	82.6 [65.9–92.1]	75.6 [60.5–87.1]
Overt EPE	31.6 [12.6–56.6]	88.5 [69.9–97.6]	66.7 [36.4–87.5]	63.9 [55.9–21.2]	64.4 [48.8–78.1]
Neurovascular bundle asymmetry	42.1 [20.3–66.5]	88.5 [69.9–97.6]	72.7 [44.8–89.7]	67.6 [58.2–75.9]	68.9 [53.4–81.8]
CLL ≥ 1.5 cm	89.5 [66.9–98.7]	57.7 [36.9–76.7]	60.7 [49.0–71.3]	88.2 [66.0–96.7]	71.1 [55.7–83.6]
SVI	52.6 [28.9–75.6]	92.3 [74.9–99.1]	83.3 [55.3–95.3]	72.7 [62.1–81.3]	75.6 [60.5–87.1]
Tumor size ≥ 14.25 mm	78.9 [54.4–94.0]	73.1 [52.2–88.4]	68.2 [52.2–80.8]	82.6 [65.9–92.1]	75.6 [60.5–87.1]
EPE grade					
EPE grade ≥ 1	89.5 [66.9–98.7]	57.7 [36.9–76.7]	60.7 [49.0–71.3]	88.2 [66.0–96.7]	71.1 [55.7–83.6]
EPE grade ≥ 2	78.9 [54.4–94.0]	73.1 [52.2–88.4]	68.2 [52.2–80.8]	82.6 [65.9–92.1]	75.6 [60.5–87.1]
EPE grade ≥ 3	31.6 [12.6–56.6]	88.5 [69.9–97.6]	66.7 [36.4–87.5]	63.9 [55.9–71.2]	64.4 [48.8–78.1]
ISUP grade					
ISUP grade ≥ 2	89.5 [66.9–98.7]	23.1 [9.0–43.7]	46.0 [39.6–52.5]	75.0 [40.4–93.0]	51.1 [35.8–66.3]
ISUP grade ≥ 3	68.4 [43.5–87.4]	61.5 [40.6–79.8]	56.5 [42.3–69.8]	72.7 [56.3–84.7]	64.4 [48.8–78.1]
ISUP grade ≥ 4	36.8 [16.3–61.6]	80.8 [60.7–93.5]	58.3 [34.4–78.9]	63.6 [54.2–72.1]	62.2 [46.5–76.2]
PET features					
Overt EPE	63.2 [38.4–83.7]	80.8 [60.7–93.5]	70.6 [50.4–85.0]	75.0 [61.8–84.8]	73.3 [58.1–85.4]
Overt SVI	26.3 [9.2–51.2]	96.2 [80.4–99.9]	83.3 [38.8–97.5]	64.1 [57.5–70.3]	66.7 [51.1–80.0]
SUVmax ≥ 13.84	52.6 [28.9–75.6]	92.3 [74.9–99.1]	83.3 [55.3–95.3]	72.7 [62.1–81.3]	75.6 [60.5–87.1]
SUVmean ≥ 7.195	57.9 [33.5–79.8]	84.6 [65.1–95.6]	73.3 [50.8–88.0]	73.3 [61.3–82.7]	73.3 [58.1–85.4]
PSMA-TV ≥ 1.395 cm^3^	94.7 [74.0–99.9]	65.4 [44.3–82.8]	66.7 [53.9–77.4]	94.4 [71.2–99.2]	77.8 [62.9–88.8]
PSA value					
logPSA ≥ 0.8717	68.4 [43.5–87.4]	61.5 [40.6–79.8]	56.5 [42.3–69.8]	72.7 [56.3–84.7]	64.4 [48.8–78.1]

The 95% confidence interval is given in brackets. EPE, extraprostatic extension; CLL, curvilinear contact length of the tumor with the capsule; SVI, seminal vesicle invasion; ISUP, International Society of Urological Pathology; SUVmax, maximum standardized uptake value; SUVmean, mean standardized uptake value; PSMA-TV, PSMA tumor volume with a threshold of 40% of the SUVmax; PSA, prostate-specific antigen; PPV, positive predictive value; NPV, negative predictive value.

**Table 3:** Univariable and multivariable logistic regression analysis for prediction of extraprostatic extension at preoperative staging.

	*p* Value	β Coefficient	Odds Ratio	95% CI
Univariable Logistic Regression Analysis				
MRI Features				
Capsular irregularity or bulge	0.001	2.32	10.2	2.5–41.4
Neurovascular bundle asymmetry	0.026	1.72	5.6	1.2–25.2
CLL ≥ 1.5 cm	0.004	2.45	11.6	2.2–60.9
SVI	0.003	2.59	13.3	2.4–73.0
Tumor size ≥ 14.25 mm	0.001	2.32	10.2	2.5–41.4
EPE grade				
EPE grade ≥ 1	0.004	2.45	11.6	2.2–60.9
EPE grade ≥ 2	0.001	2.32	10.2	2.5–41.4
ISUP grade				
ISUP grade ≥ 3	0.051	1.24	3.5	1.0–12.1
PET features				
Overt EPE	0.004	1.97	7.2	1.9–27.7
SUVmax ≥ 13.84	0.003	2.59	13.3	2.4–73.0
SUVmean ≥ 7.195	0.005	2.02	7.6	1.9–30.7
PSMA-TV ≥ 1.395 cm^3^	0.001	3.53	34.0	3.9–297.7
Multivariable logistic regression analysis				
EPE grade and ISUP grade				
EPE grade ≥ 2	0.001	1.16	3.2	1.6–6.4
ISUP grade ≥ 3	0.177	0.49	1.6	0.8–3.3
Intercept	0.266	0.67		
EPE grade and ISUP grade and tumor size				
Tumor size ≥ 14.25 mm	0.001	1.42	4.1	1.7–9.8
EPE grade ≥ 2	0.130	0.64	1.9	0.8–4.3
ISUP grade ≥ 3	0.026	0.97	2.7	1.1–6.3
Intercept	0.216	0.62		
PET features only				
Overt EPE on PET	0.583	0.24	1.3	1.5–3.1
SUVmax ≥ 13.84	0.022	1.38	4.0	1.2–12.9
PSMA-TV ≥ 1.395 cm^3^	0.005	1.83	6.2	1.8–21.9
Intercept	0.503	−0.43		
EPE grade and SUVmax				
EPE grade ≥ 2	0.007	1.06	2.9	1.3–6.3
SUVmax ≥ 13.84	0.014	1.18	3.3	1.3–8.3
Intercept	0.693	0.19		
EPE grade and PSMA-TV				
EPE grade ≥ 2	0.075	0.74	2.1	0.9–4.7
PSMA-TV ≥ 1.395 cm^3^	0.010	1.48	4.4	1.4–13.5
Intercept	0.069	−1.03		
EPE grade and ISUP grade and SUVmax				
EPE grade ≥ 2	0.007	1.06	2.9	1.3–6.3
ISUP grade ≥ 3	0.426	0.32	1.4	0.6–3.0
SUVmax ≥ 13.84	0.014	1.18	3.3	1.3–8.3
Intercept	0.693	0.19		
EPE grade and ISUP grade and PSMA-TV				
EPE grade ≥ 2	0.106	0.70	2.0	0.86–4.8
ISUP grade ≥ 3	0.061	0.78	2.2	1.0–4.9
PSMA-TV ≥ 1.395 cm^3^	0.001	1.86	6.4	2.0–20.0
Intercept	0.044	−1.15		
EPE grade and ISUP grade and Tumor size and SUVmax				
EPE grade ≥ 2	0.135	0.66	1.9	0.8–4.6
ISUP grade ≥ 3	0.089	0.79	2.2	0.9–5.5
Tumor size ≥ 14.25 mm	0.006	1.30	3.7	1.5–9.1
SUVmax ≥ 13.84	0.046	0.97	2.6	1.0–6.8
Intercept	0.987	−0.008		

CLL, curvilinear contact length of the tumor with the capsule; SVI, seminal vesicle invasion; EPE, extraprostatic extension; ISUP, International Society of Urological Pathology; SUVmax, maximum standardized uptake value; SUVmean, mean standardized uptake value; PSMA-TV, PSMA tumor volume with a threshold of 40% of the SUVmax; CI, confidence interval.

## Data Availability

The data presented in this study are available on request from the corresponding author after completion of the data exchange agreement due to institutional regulations.
